# Transposable Element-Derived miR-28-5p and miR-708-5p: Exploring Potential Roles in Lung Cancer

**DOI:** 10.3390/ncrna11060081

**Published:** 2025-12-18

**Authors:** Sergiu Chira, Cornelia Braicu, Stefan Strilciuc, George A. Calin, Ioana Berindan-Neagoe

**Affiliations:** 1 Department of Genomics, Medfuture Institute for Biomedical Research, Iuliu Hațieganu University of Medicine and Pharmacy, 400337 Cluj-Napoca, Romania; sergiu.chira@umfcluj.ro (S.C.); stefan.strilciuc@medfuture.ro (S.S.); ioana.neagoe@umfcluj.ro (I.B.-N.); 2 Department of Cancer Biology, The University of Texas MD Anderson Cancer Center, 1515 Holcombe Boulevard, Unit 422, Houston, TX 77030, USA; gcalin@mdanderson.org; 3 Department of Translational Molecular Pathology, The University of Texas MD Anderson Cancer Center, 1515 Holcombe Boulevard, Unit 422, Houston, TX 77030, USA; 4 Center for RNA Interference and Non-Coding RNAs, The University of Texas MD Anderson Cancer Center, Houston, TX 77030, USA; 5 Doctoral School, Iuliu Hatieganu University of Medicine and Pharmacy, 400337 Cluj-Napoca, Romania; 6 Academy of Medical Sciences, 030167 Bucharest, Romania

**Keywords:** transposable elements, mir-28, mir-708, lung cancer

## Abstract

**Background:** Transposable elements are normally silenced by epigenetic mechanisms; however, during malignant transformation, epigenetic alterations enable transposons to produce functional molecules like miRNAs. Among these, LINE-2 (L2) elements can generate miRNAs capable of regulating key genes, including tumor suppressors. Two L2-derived miRNAs, miR-28 and miR-708, have been linked to lung cancer, yet the mechanisms underlying their dysregulation remain poorly understood. Our study reveals how genomic context contributes to aberrant gene expression through comprehensive bioinformatic analyses. **Methods:** Using bioinformatics analysis, we evaluated the expression of miR-28 and miR-708 in lung adenocarcinoma (LUAD) and lung squamous cell carcinoma (LUSC) datasets from TCGA. Further, we assessed the expression and methylation status of miR-28 and miR-708 host genes, *LPP* and *TENM4*, respectively, using computational tools. Finaly, we searched for potential candidate tumor suppressor genes targeted by miR-28 and miR-708, which are downregulated in LUAD and LUSC. **Results:** We found that intragenic L2-derived miR-28 and miR-708 are significantly upregulated in LUAD and LUSC. While *TENM4* gene also displays a marked increase in expression in LUAD and LUSC, in tumor versus normal tissue, this difference is less obvious for the *LPP* gene. We suggest that such dysregulations in expression might be linked to specific methylation patterns of their genomic locations. Furthermore, we emphasize that miR-28 and miR-708 might contribute to lung cancer pathogenesis by targeting key tumor suppressor genes. **Conclusions:** Alterations in the methylation status of L2-miRNAs genomic loci might result in elevated levels of miRNAs and subsequent targeting of tumor suppressor genes with potential implications in lung cancer pathogenesis.

## 1. Introduction

Transposable elements (TE), comprising a significant fraction of our genome, are well-known drivers of evolution. As their activity has shaped our DNA throughout the primate evolution [1], a more complex role besides being simple “jumping genes” can be associated with transposons [2]. Firstly, by integrating into new genomic locations, transposons can generate double strand breaks and inherently lead to genomic instability [3]. These mobile elements carry sequences with regulatory functions, which in turn can modulate the activity of neighboring genomic regions by diverse mechanisms, such as transcription activation, alternative splicing or premature polyadenylation [4]. To counteract the deleterious effects that mobile elements might pose, cells have adapted mechanisms to limit their activity, primarily by epigenetic modifications, such as methylation of TE-encoded genomic loci [5]. However, in the early stages of tumorigenesis, cells undergo a genome-wide hypomethylation [6,7], which leads to reactivation of the TE, an event associated with increased genomic instability [8] and expression of oncogenes [9]. Besides the ability to actively transcribe oncogenes, TE might also alter gene expression at the post-transcriptional level by providing RNA transcripts with regulatory functions, such as microRNAs (miRNA) [4,10]. miRNAs are short transcripts, 18–22 nucleotide-long small non-coding RNAs that regulate gene expression post-transcriptionally [11] through binding of messenger RNAs (mRNAs) and recruiting protein complexes with catalytic activity, an event that leads to mRNA degradation and reduced protein synthesis. Because miRNA binding relies on imperfect sequence complementarity, a single miRNA can regulate the expression of thousands of genes, and conversely, a single mRNA can be targeted by multiple miRNAs [12]. This property of miRNAs gives rise to complex regulatory networks that can be disrupted in malignant cells, thereby contributing to carcinogenesis and tumor progression [13,14,15]. TEs are closely connected to the miRNA regulatory network, serving as sources of miRNA sequences or providing regulatory elements that influence miRNA expression. This is the case of the Long Interspersed Nuclear Elements 2 (LINE2), which are the origin of at least 19 miRNAs [4]. Two of them, miR-28-5p and miR-708-5p, are of particular importance, as their aberrant expression is suggested to be linked to various cancers, including lung cancer [16], acting both as tumor-promoting (oncomiRs) or tumor suppressor miRNAs depending on the tumor type (Table 1). However, the mechanisms underlying their altered expression remain unclear. Using a bioinformatics approach, we aim to investigate whether an aberrant expression of miR-28 and miR-708 might be linked to epigenetic changes in the LINE2 genomic loci that encodes them and if such changes might contribute to lung adenocarcinoma (LUAD) and lung squamous cell carcinoma (LUSC) pathogenesis, the two major subtypes of non-small-cell lung carcinoma (NSCLC).

## 2. Results

### 2.1. Coding Sequences for miR-28-5p and miR-708-5p Are Located Within Intronic LINE-2 Transposons

**Figure 1:**
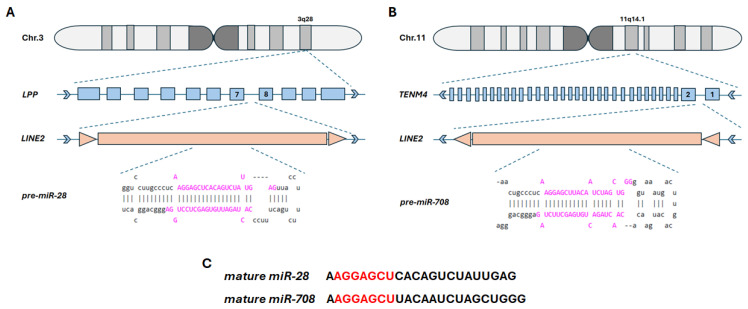
Schematic representation of genomic locations for miR-28-5p and miR-708-5p. (**A**). The encoding sequence for pre-miR-28-5p overlaps with the LINE-2 transposon, which is located within the intronic region of exon 7 and 8 (blue boxes) of the *LPP* gene on chromosome 3, band q28 (3q28). (**B**). The pre-miR-708-5p sequence is also embedded in a LINE-2 transposon, located in the intronic region between exon 1 and 2 (blue boxes) of the *TENM4* gene on chromosome 11, band q14.1 (11q14.1). (**C**). Sequences of the mature miR-28-5p and miR-708-5p, in which the seed region is indicated in red. Pre-miR-28-5p and pre-miR-708-5p sequences are illustrated in their stem-loop conformation, and the mature sequence bases are highlighted in pink font. LINE-2 transposon is represented in orange boxes flanked by two orange triangles (direct repeats). Blue arrow heads to the right indicate the positive strand of the genomic DNA, while blue arrow heads to the left indicate the minus strand of the genomic DNA.

To identify the genomic loci of miR-28-5p and miR-708-5p coding sequences, we mapped their locations within the human genome hg38 using Genome Browser [33]. We found the coding sequence for miR-28-5p to be located on plus strand of chromosome 3 (chr3:188,688,781–188,688,866), within the seventh intron of LIM containing preferred translocation partner in lipoma (*LPP*) gene. For miR-708-5p, the coding sequence is positioned on the minus strand of chromosome 11 (chr11:79,402,022–79,402,109), embedded in the first intron of the teneurin transmembrane protein 4 (*TENM4*). Both miRNA-coding sequences overlap with L2c repeats of the LINE-2 elements (Figure 1A,B), as previously reported [4]. miR-28-5p and miR-708-5p share the same seed sequence, spanning from the 2nd to the 8th nucleotide base of the mature miR sequences, though the 3′ sequences differ between the two miRNAs (Figure 1C).

### 2.2. In Silico Expression Analysis of miR-28-5p and miR-708-5p, and Their Host Genes in LUAD and LUSC

**Figure 2:**
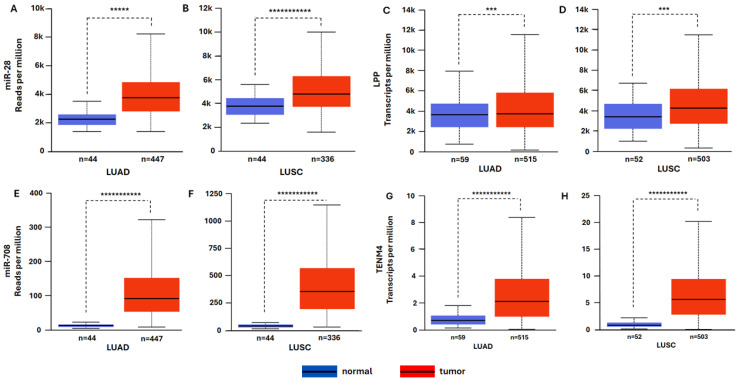
Expression of miR-28-5p and miR-708-5p, and their host genes *LPP* and *TENM4*, in LUAD and LUSC. Expression analysis was performed on lung adenocarcinoma (LUAD) and lung squamous cell carcinoma (LUSC) datasets from TCGA using UALCAN software. Expression of miR-28-5p in normal and primary tumor tissues from patients with LUAD (**A**) and LUSC (**B**). Expression of *LPP* gene in normal and primary tumor tissues from patients with LUAD (**C**) and LUSC (**D**). Expression of miR-708-5p in normal and primary tumor tissues from patients with LUAD (**E**) and LUSC (**F**). Expression of *TENM4* gene in normal and primary tumor tissues from patients with LUAD (**G**) and LUSC (**H**). Blue bars illustrate expression in normal tissues, while red bars represent expression in tumor tissues. Number of samples used for analysis are indicated under each bar. Levels of expression are indicated as reads per million for the miRNA sequences, and transcripts per million for the gene transcripts (reads per million is used to normalize RNA-seq data for small RNAs, and transcript per million for normalization of RNA-seq data for mRNA). *** *p*-value ≤ 0.001, ***** *p*-value ≤ 0.00001, *********** *p*-value ≤ 1 × 10^−11^.

To further investigate if miR-28-5p-5p and miR-708-5p-5p are aberrantly co-expressed with the *LPP* and *TENM4* genes that harbor them, we analyzed their expression in LUAD and LUSC datasets from TCGA using the University of Alabama at Birmingham Cancer data analysis portal UALCAN [34] (https://ualcan.path.uab.edu/analysis.html, accessed on 20 October 2025). This analysis highlighted that both miR-28-5p and miR-708-5p are overexpressed in tumor tissue when compared to normal tissue, in LUAD and LUSC (Figure 2A,B,E,F). However, its host gene, *LPP*, does not display significantly elevated expression in tumor tissues, as indicated in Figure 2C,D. However, the *TENM4* gene transcript exhibits a pronounced increase in expression in tumor tissue compared to normal tissue, in both LUAD and LUSC (Figure 2E–H).

### 2.3. In Silico Methylation Analysis of LPP and TENM4 Genes in LUAD and LUSC

**Figure 3:**
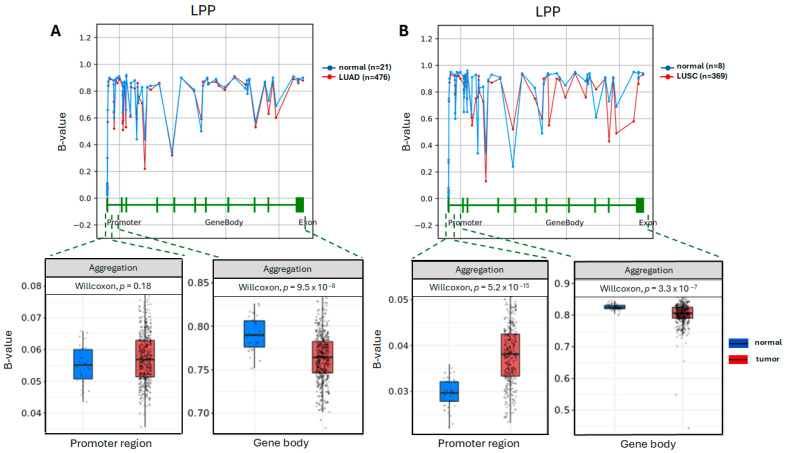

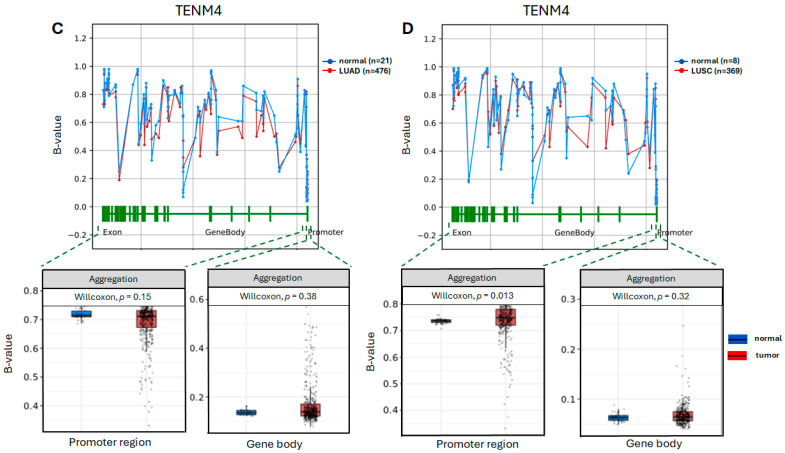
Methylation analysis of the *LPP* and *TENM4* genes in LUAD and LUSC. Differential methylation of the *LPP* gene in normal and tumor tissue in LUAD (**A**) and LUSC (**B**). Differential methylation of the *TENM4* gene in normal and tumor tissue in LUAD (**C**) and LUSC (**D**). The methylation distribution throughout the *LPP* and *TENM4* genes is represented as blue dotted lines (normal tissue) and red dotted lines (tumor tissue) in the upper part of each panel. Number of samples used for analysis is indicated in the right corner of the panels. At the bottom of the panels, genes are represented as horizontal green lines, while the exons are indicated as vertical green bars. For the promoter region and gene bodies, statistical significance of methylation is plotted in blue (normal tissue) and red (tumor tissue) bars, and the Wilcoxon rank *p*-value is indicated in the upper part of each panel. Methylation is indicated as B-value on the y-axis. Aggregation indicates the mean methylation for all individual CpG sites selected for that region in all samples analyzed. LUAD—lung adenocarcinoma; LUSC—lung squamous cell carcinoma.

As retrotransposons activation is associated with hypomethylation of the genomic loci that harbors them [35,36], we looked further to see if the methylation status of *LPP* and *TENM4* genomic loci is altered in LUAD and LUSC. For this, we used the OncoDB v2.0 online tool [37], which uses data from TCGA consortium for multi-omics analyses of human cancers. The LUAD and LUSC datasets were used for methylation analysis of *LPP* and *TENM4* genes. To further investigate the statistical significance of the methylation level on specific regions, such as CpG islands near the 5′-end of the gene where the promoter resides, and throughout the gene’s body, we used the Shiny Methylation Analysis Research Tool online SMART [38] (http://www.bioinfo-zs.com/smartapp/, accessed on 20 October 2025). This software allows selection of individual or multiple methylation probes that can be used to evaluate the methylation level for a particular gene. The analysis highlighted that for the *LPP* gene, a varying degree of methylation depending on the position in the gene can be observed (upper panel of Figure 3A); however, there is no statistical difference between tumor and normal tissue in LUAD at the promoter region (bottom panel of Figure 3A). For the gene body of *LPP*, a significant difference can be seen, suggesting a lower level of methylation in the tumor tissue compared to normal tissue (bottom panel of Figure 3A).

In LUSC, a statistically higher level of methylation can be observed for the promoter region of the *LPP* gene in tumor tissue, though the B-value for both tumor and normal is below 0.05 (bottom panel of Figure 3B), meaning that less than 5% of all samples included in analysis are methylated at this region in LUSC. This points out that *LPP* promoter region is hypomethylated in both normal and tumor tissue. The reminder of the *LPP* gene is statistically hypomethylated in tumor tissue compared to normal tissue, in both LUAD and LUSC (bottom panels of Figure 3A,B). As in the case of *LPP*, the *TENM4* gene also displays a varying degree of methylation depending on the region, and tumor vs. normal tissue, with no statistical significance of the methylation sites in the promoter region for normal vs. tumor tissue in both LUAD and LUSC (bottom panels of Figure 3C,D). A slight increase in the overall methylation throughout the *TENM4* gene body can be seen in tumor vs. normal tissue in LUSC (*p*-value 0.013), whereas for LUAD, no statistical difference was observed (upper panels of Figure 3C,D).

**Figure 4:**
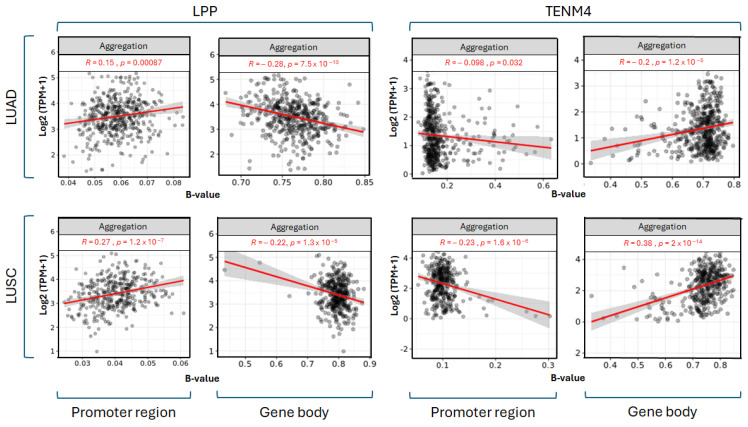
Pearson statistical correlation of expression and methylation in the promoter region and gene body of the *LPP* and *TENM4* genes in LUAD and LUSC. Level of expression is indicated on y-axis as Log2(TPM + 1), while methylation is represented on the x-axis as B-value. The correlation coefficient (R) and statistical significance (*p*-values) are indicated in red at the top of each panel. Aggregation indicates the mean methylation for all individual CpG sites selected for that region in all samples analyzed. LUAD—lung adenocarcinoma; LUSC—lung squamous cell carcinoma.

For correlating methylation of the analyzed regions with expression of the host genes, we used the SMART online tool, with Pearson statistical method for correlation analysis [38]. The *LPP* gene shows a differential correlation depending on the region analyzed, with the promoter region displaying a positive correlation of *LPP* expression and methylation, while the gene body indicates a negative correlation. This pattern can be seen in both LUAD and LUSC for the *LPP* gene (Figure 4). For the *TENM4* gene, the correlation is quite opposite to *LPP*, with the promoter region having a negative correlation of expression and methylation, while the remainder of the *TENM4* gene has a positive correlation in LUAD and LUSC (Figure 4).

### 2.4. miR-28-5p and miR-708-5p Might Regulate a Common Set of Tumor Suppressor Genes in LUAD and LUSC

**Figure 5:**
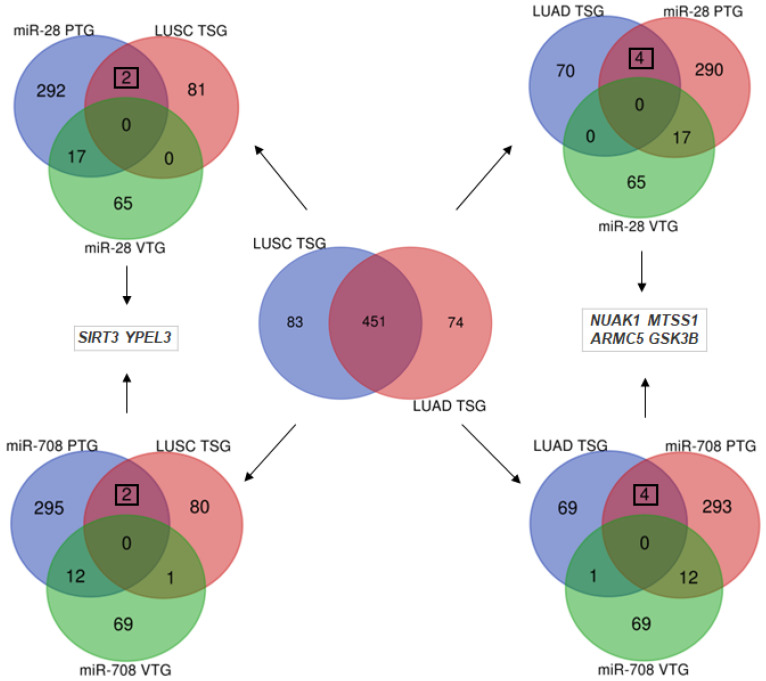
Venn diagram analysis of tumor suppressor genes targeted by miR-28-5p and miR-708-5p in LUAD and LUSC. The tumor suppressor genes (TSGs) unique for LUSC and for LUAD have been further used to highlight TSGs that are targeted by miR-28-5p and miR-708-5p by intersection with predicted targets genes (PTG) and validated target genes (VTGs) of miR-28-5p and miR-708-5p, respectively. Numbers represent the genes in each list, and numbers in squares represent the genes that are potentially targeted by both miR-28-5p and miR-708-5p in LUAD and LUSC. Genes symbols are indicated in italic. LUAD—lung adenocarcinoma; LUSC—lung squamous cell carcinoma.

To determine whether miR-28-5p and miR-708-5p might target tumor suppressor genes (TSGs) in LUAD and LUSC, and if differences can be highlighted, we downloaded from the Tumor Suppressor Gene Database [39] a list of 525 TSGs downregulated in LUAD, and a list of 534 TSGs downregulated in LUSC. Common and unique downregulated TSGs in LUAD and LUSC were further inferred by Venn diagram (Figure 5). In the next step, we looked if among the unique TSGs downregulated in LUAD and LUSC, potential target genes for miR-28-5p and miR-708-5p can be identified. We retrieved the predicted target genes (PTGs) from miRDB [40] and the validated target genes (VTGs) from miRTarBase [41] and performed intersections with the downregulated TSGs in LUAD and LUSC. This analysis highlighted that miR-28-5p and miR-708-5p might target different TSGs in LUAD when compared to LUSC (Figure 5). In LUSC, two TSGs, namely Sirtuin 3 (*SIRT3*) and Yippee Like 3 (*YPEL3*), were found to be potential targets for miR-28-5p and miR-708-5p. In LUAD, these two miRNAs have as potential targets the NUAK Family SNF1-Like Kinase 1 (*NUAK1*), the Metastasis Suppressor Protein 1 (*MTSS1*), Armadillo Repeat-Containing Protein 5 (*ARMC5*) and the Glycogen Synthase-3 Beta (*GSK3B*). As depicted in Figure 5, the six TSGs have not yet been validated as targets for miR-28-5p and miR-708-5p, because they are not among the genes found in the miRTarBase database.

### 2.5. LPP and TENM4 Protein Expression in LUAD and LUSC

**Figure 6:**
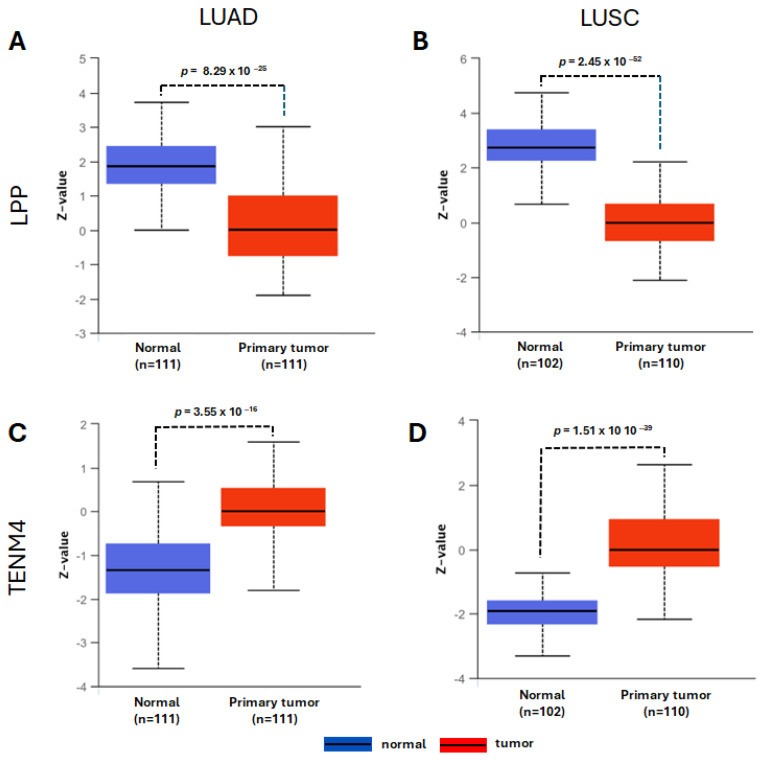
Protein expression levels of LPP and TENM4 in LUAD and LUSC. Level of LPP protein in LUAD (**A**) and LUSC (**B**), and level of TENM4 protein in LUAD (**C**) and LUSC (**D**). Plots were generated using UALCAN software. Blue bars represent protein levels in normal tissues, and red bars for the tumor tissues. The number of samples used for analysis is indicated under bars. Z-values represent the standard deviations from mean across samples for LUAD and LUSC. Spectral count ratio values from Clinical Proteomics Tumor Analysis Consortium (CPTAC) were first normalized within each sample, then normalized across samples.

In the next step, we wondered if the elevated expression of LPP and TENM4 transcripts further translate into high levels of their encoded proteins. For this, we used the integrated Clinical Proteomic Tumor Analysis Consortium (CPTAC) database implemented in UALCAN online tool (https://ualcan.path.uab.edu/analysis.html, accessed on 20 October 2025). The analysis highlighted an increase in TENM4 protein in tumor tissue compared to normal tissue, in accordance with an elevated level of TENM4 transcript, while the level of LPP protein is inversely correlated with the LPP transcript level in tumor versus normal tissue, in both LUAD and LUSC (Figure 6). These results point that LPP protein might function as a tumor suppressor rather than an oncoprotein, and that its expression is tightly regulated post-transcriptionally in tumor tissue compared to normal tissue. In contrast, TENM4 might function as an oncoprotein, as both the transcript and protein display an increase in expression in tumor versus normal tissue, with potential implications in LUAD and LUSC pathogenesis.

## 3. Discussion

One important mechanism of gene regulation in cells is mediated by miRNAs. These small non-coding transcripts bind to messenger RNAs (mRNAs) and recruit protein complexes with catalytic activity, leading to mRNA degradation and reduced protein synthesis. Because miRNA binding relies on imperfect sequence complementarity, a single miRNA can regulate the expression of thousands of genes, and conversely, a single mRNA can be targeted by multiple miRNAs [12]. This property of miRNAs gives rise to complex regulatory networks that can be disrupted in malignant cells, thereby contributing to carcinogenesis and tumor progression [13,14,15]. TEs are closely connected to the miRNA regulatory network, serving as sources of miRNA sequences or providing regulatory elements that influence miRNA expression. Through these contributions, TEs can shape gene regulatory networks, affecting key cellular processes [4]. In the present study, we focused on two transposon-derived miRNAs, miR-28-5p and miR-708-5p, which have been found to be linked to various human cancers, functioning both as tumor suppressors and tumor-promoting miRNAs (oncomiRs) depending on the tumor type (Table 1).

These two miRNAs have been shown to be involved in major processes related to tumor growth, proliferation, metastasis, apoptosis or cancer stem cell self-renewal (Table 1 and references within). Our analysis aims to investigate whether an aberrant expression of miR-28-5p and miR-708-5p might be a consequence of alterations in the methylation status of their host genes, the *LPP* and *TENM4* genes, and to assess any potential implications such changes might have in NSCLC carcinogenesis. In the first step, we investigated the expression levels of miR-28 and miR-708 in the two major subtypes of NSCLC, LUAD and LUSC. For this, we used the UALCAN tool [34], which enables analysis of the TCGA samples database [42]. This analysis highlighted an upregulation of miR-28-5p and miR-708-5p (tumor versus normal tissue) in both LUAD and LUSC, with miR-708-5p displaying the most significant upregulation (Figure 2A,B,E,F). Next, we wondered if the observed elevated expression of miR-28-5p and miR-708-5p might be linked to their genomic context. The coding sequence for miR-28-5p was mapped in the seventh intron of the *LPP* gene, located on chromosome 3, and within the first intron of the *TENM4* gene on chromosome 11 for miR-708-5p (Figure 1). This is not surprising, as TE are mostly distributed in intronic regions of the genes [43], including those that encode miRNAs in their sequences [10]. As hypomethylation is a hallmark of cancer cells [6,7] and this further correlates with increased transposons activity [8], we investigated the methylation status of the *LPP* and *TENM4* genes that harbor L2-derived miR-28-5p and miR-708-5p, respectively. The analysis shows that the *LPP* gene has differential degrees of methylation depending on the region analyzed. For the promoter region, which is enriched in CpG islands, no statistical difference could be observed in tumor vs. normal tissue in LUAD (Figure 3A), though in LUSC, the promoter region has a higher degree of methylation in tumor vs. normal tissue (Figure 3B). The gene body of *LPP* displays a significantly lower level of methylation in tumor vs. normal tissue in both LUAD and LUSC (Figure 3A,B). These differences seen in the gene body of *LPP* are mostly enriched in intronic regions (upper panels of Figure 3A,B). For *TENM4*, no significant differences can be seen in the overall methylation status of the gene body in LUAD and LUSC (bottom panels of Figure 3C,D). However, methylation varies between regions of the *TENM4* gene body, with the first half (towards the promoter region) comprised mostly of intronic sequences, displaying the highest variations in methylation between tumor and normal tissues (upper panels of Figure 3C,D). The promoter region of *TENM4* exhibits no significant differences in methylation (bottom panels of Figure 3C,D), suggesting that both tumor and normal tissue have relatively similar activities for this domain of the *TENM4* gene in LUAD and LUSC. To better understand if the observed differences and similarities in methylation have a statistical significance regarding the expression of the *LPP* and *TENM4* genes, we inferred the Pearson correlation between methylation and expression using the SMART software [38]. For *LPP*, we observed a positive correlation between methylation and expression for the promoter region, and a negative correlation for the gene body, in both LUAD and LUSC (Figure 4). In contrast, *TENM4* exhibits a negative correlation for the promoter region, while the gene body displays a positive correlation between methylation and expression in LUAD and LUSC (Figure 4). These observations are in contradiction with the common belief of methylation, in which DNA methylation and transcription have a negative correlation, meaning that a high methylation status is associated with a low expression of genes, and vice versa. However, this is not necessarily universal, and a positive correlation with expression is quite common for gene bodies, where it is associated with an increase in gene expression levels [44], and this propriety might be a feature of proliferative tissues and cell lines [45]. However, the mechanisms by which methylation increases gene expression are not fully understood, and it was suggested that it may contribute to an open state of chromatin structure and increased accessibility of transcription factors to the genomic DNA [44]. However, our analysis also points to a positive correlation between promoter methylation and expression, as in the case of *LPP* gene (Figure 4). This further portrays the spatiotemporal control of methylation in gene expression regulation, as highlighted by a pan-cancer analysis of methylation sites in tumor genomes, where it was shown that approximately one third of the promoter regions analyzed show a positive correlation of methylation and expression [46]. In our study, a positive correlation of methylation and expression in the promoter region, and a negative correlation of the gene body for *LPP*, is associated with an increase in the expression of intragenic region as suggested by miR-28-5p level in tumor tissues, and to a lower extent in *LPP* expression in LUAD and LUSC (Figure 2A–D). For *TENM4* gene, the negative correlation of promoter methylation and expression, together with a positive correlation between gene body methylation and expression, is linked to a marked increase in expression of both *TENM4* gene and the intragenic miR-708-5p in LUAD and LUSC (Figure 2E–H). These observations suggest that differential methylation between different regions of the same gene might reflect in distinct expression patterns, highlighting the diversity of gene expression regulation. However, it should be noted that sample size in the input data used for expression analysis and methylation analysis might differ, and other correlations should not be ruled out.

The LPP protein has been shown to be involved in cytoskeleton organization and cell adhesion [46,47], though it may also function as a transcription activator [48]. Loss of LPP function is associated with tumor cell dissemination in mouse orthotopic model of lung adenocarcinoma cell line PC14PE6 [49], pointing to a potential tumor suppressor role. However, LPP might also function as an oncogene by promoting metastasis, as suggested by studies on breast cancer cells in mice models [50]. Therefore, the precise role of LPP in LUAD and LUSC remains to be investigated. The TENM4 function has initially been described in neural cell development and function [51] and data for a potential oncogenic role is quite thin, though some studies point out that it may be a trait of cancer cell stemness and invasiveness of highly aggressive tumors, such as triple-negative breast cancer [52]. Data presented in this paper shows a marked increase in transcript level in tumor tissue when compared to normal tissue (Figure 2G,H), suggesting an oncogenic role of TENM4 in LUAD and LUSC. While the TENM4 transcript overexpression (Figure 2G,H) is also associated with an increase in TENM4 protein in tumor tissue (Figure 6C,D), the relative increase in LPP transcript level in tumor tissue (Figure 2C,D) relates to a significant decrease in LPP protein (Figure 6A,B). This might suggest an oncogenic role only for TENM4 while the LPP might function as a tumor suppressor, as suggested by other studies [49]. This suggests that the *LPP* gene is tightly regulated post-transcriptionally in both LUAD and LUSC, possibly by the fact that *LPP* harbors the coding sequence for miR-28, which is upregulated in tumors (Figure 2A,B). However, this also might apply for *TENM4* gene, as miR-708 is overexpressed in tumors (Figure 2E,F), though the TENM4 protein level is also higher in tumors (Figure 6C,D). Therefore, other mechanisms beside miR-28 silencing of *LPP* in tumors should be considered. As the TCGA expression data also highlighted an increase in miR-28-5p and miR-708-5p expression levels (Figure 2A,B,E,F), we next wondered if other TSGs might be among the targets for the two miRNAs. For this, we retrieved all TSGs from Tumor Suppressor Gene Database for LUAD and LUSC [39] and identified common TSGs in LUAD and LUSC by intersection with predicted and validated target of miR-28-5p and miR-708-5p. Our analysis led to a shortlist of two target TSGs in LUSC, namely *SIRT3* and *YELP3* genes, and three targeted TSGs in LUAD, *NUAK1*, *MTSS1*, *ARMC5* and *GSK3B* (Figure 5).

The tumor suppressor function of SIRT3 gene has been recently described in NSCLC, where it has been shown to inhibit cell proliferation and increase ROS levels in the malignant cells [53], and to impair tumor development in murine lung cancer models [54], suggesting an important tumor suppressor gene. Also, *YPEL3*, a downstream effector of p53, has been suggested to function as a tumor suppressor by inducing senescence in breast tumor cells [55], and by suppressing epithelial to mesenchymal transition (EMT) and tumor metastasis by targeting Wnt/β-catenin signaling pathway in nasopharyngeal carcinoma cells [56]. However, its tumor suppressor potential in lung cancer has not yet been uncovered. For the miR-28-5p and miR-708-5p targets in LUAD, data highlight *NUAK1* as an oncogenic gene rather than tumor suppressor, being associated with a poor prognosis in NSCLC patients [57,58]. The mechanisms by which *NUAK1* promotes tumor progression are various and have been linked to regulation of metalloprotein-2 and -9 (MMP-2, MMP-9) [57], and therefore metastasis, or to mTOR-regulated glucose metabolism reprogramming by suppressing p53 activity [58]. In esophageal squamous cell carcinoma, it regulates metastasis by activating JNK/c-Jun/Slug signaling pathway [59]. Also, *NUAK1* can provide the tumor cell with resistance to chemotherapy by upregulating the STAT/GLI1/SOX2 signaling pathway, as indicated by a recent study on gastric cancer [60]. In contrast, the *MTSS1* gene has been shown to exhibit both tumor suppression and tumor-promoting function in lung cancer, depending on the tumor subtype. In LUAD, *MTSS1* enhances proliferation and invasion [61], and immune surveillance evasion [62], while in LUSC, *MTSS1* acts as a tumor suppressor by inhibition of these processes [61].

For the other potential targets of miR-28-5p and miR-708-5p, *ARMC5* and *GSK3B*, data is rather thin. A positive correlation between *GSK3B* expression and clinical outcome of NSCLC patients has been suggested, patients with higher expression level of *GSK3B* displaying the worse prognosis [63]. The mechanisms by which *GSK3B* might be playing tumor-promoting function remain unclear, though other studies point to *GSK3B* as a regulator of metabolic reprogramming in cancer [64].

## 4. Materials and Methods

### 4.1. miR-28-5p and miR-708-5p Sequence Analysis

The genomic loci of miR-28-5p and miR-708-5p were identified using the UCSC Genome Browser (GRCh38/hg38) [65]. Overlap with LINE-2 elements was confirmed using the Repeat Masker annotations [66]. Mature sequences of miR-28-5p and miR-708-5p were retrieved from miRbase [67], and their seed sequences were identified and highlighted to assess sequence conservation.

### 4.2. In Silico Expression Analysis in NSCLC

Expression of miR-28-5p, miR-708-5p, and their host genes (*LPP* and *TENM4*) were assessed in non-small-cell lung cancer (NSCLC) datasets from The Cancer Genome Atlas (TCGA) for lung adenocarcinoma (LUAD) and lung squamous cell carcinoma (LUSC) cohorts. Analyses were performed using the University of Alabama at Birmingham Cancer data analysis portal online tool UALCAN [34] (https://ualcan.path.uab.edu/analysis.html, accessed on 20 October 2025), which provides interactive visualization of TCGA transcriptome data. Normal versus tumor expression levels were compared, and expression was represented as reads per million for miRNAs and transcripts per million for host genes. Statistical significance was determined by UALCAN’s built-in statistical testing (Student’s *t*-test).

### 4.3. DNA Methylation Analysis

Methylation profiles of the *LPP* and *TENM4* loci in LUAD and LUSC were evaluated using OncoDB [37], a multi-omics analysis platform that integrates TCGA data. To assess methylation at specific CpG sites, particularly at promoter-associated CpG islands and across gene bodies, we employed the Shiny Methylation Analysis Research Tool online SMART online tool [38] (http://www.bioinfo-zs.com/smartapp/, accessed on 20 October 2025). SMART enables probe-level analysis of TCGA methylation data and allows regional comparisons. For each gene, methylation levels were expressed as beta (B) values, ranging from 0 (indicating no methylation) to 1 (indicating complete methylation). Differential methylation between tumor and normal tissues was assessed using Wilcoxon rank-sum test, with statistical significance indicated in figures.

### 4.4. Correlation Between Gene Expression and DNA Methylation

Correlation between methylation levels in the promoter region (CpG islands) and gene body, and expression of *LPP* and *TENM4*, was determined using Pearson correlation analysis implemented in SMART. Expression data [Log2(TPM + 1)] and methylation B-values were plotted, and the correlation coefficient (R) along with *p*-values were calculated for LUAD and LUSC datasets separately.

### 4.5. Identification of Candidate Tumor Suppressor Gene Targets of miR-28-5p and miR-708-5p

Lists of downregulated TSGs were obtained from the Tumor Suppressor Gene Database [39]. Common and subtype-specific TSGs were identified using Venn diagram analysis. Predicted target genes (PTGs) of miR-28-5p and miR-708-5p were retrieved from miRDB [40], and validated target genes (VTGs) from miRTarBase [41]. Intersections between PTGs/VTGs and unique downregulated TSGs in LUAD and LUSC were used to identify potential subtype-specific TSGs which are targeted by miR-28-5p and miR-708-5p.

### 4.6. Protein Expression Analysis

LPP and TENM4 protein levels in LUAD and LUSC were assessed in the Clinical Proteomic Tumor Analysis Consortium (CPTAC) integrated in UALCAN online tool [34]. Total LPP and TENM4 protein levels are expressed as Z-values, which represent the standard deviation from mean across samples for LUAD and LUSC. Spectral count ratio values from CPTAC were first normalized within each sample, then normalized across samples.

## 5. Conclusions

Transposable elements are important players in human genome evolution, function and regulation. Their mobilization can result in diverse effects on the genomic DNA; however, transposons, such as LINE2 (L2), can have an impact on the human transcriptome by providing RNA transcripts with regulatory function, such as miRNAs. In this study, we show that L2-derived miR-28 and miR-708 are overexpressed in LUAD and LUSC, likely driven by differential methylation of their genomic loci. This deregulation may facilitate repression of tumor suppressor genes, linking transposable element activation to oncogenic pathways in NSCLC. Our findings highlight TE-derived miRNAs as overlooked contributors to lung cancer biology, with potential utility as biomarkers of epigenetic deregulation. However, it should be noted that our analysis is based on computational tools to highlight a potential link between L2 retrotransposons–miRNAs–tumor suppressor gene–lung cancer. Experimental validation in a cohort of patients with LUAD and LUSC for establishing a direct connection between L2 transposons and LUAD and LUSC pathogenesis is needed. In addition, an evaluation of L2 methylation status and transcript expression is equally important to link these transposons to lung cancer. Our study provides a critical first step toward understanding the potential roles of these two miRNAs and lays the scientific groundwork for future mechanistic and experimental validation.

## Figures and Tables

**Table 1:** Differential expression and functional roles of miR-28-5p and miR-708-5p in various cancer types.

miRNA	Cancer Type	Expression (TT vs. NT)	Functional Role	Regulated Processes	Reference
miR-28-5p	NSCLC	up	oncomiR	Proliferation	[16]
	Glioblastoma	up	oncomiR	Tumor sphere formation, cell viability and proliferation	[17]
	Ovarian cancer	up	oncomiR	Cell cycle, apoptosis	[18]
	Gastric cancer	up	oncomiR	Proliferation and invasion	[19]
	Breast cancer	down	tumor suppressor	Migration	[20]
	Hepatocellular carcinoma	down	tumor suppressor	Cancer cell stemness, treatment resistance	[21]
	Gastric cancer	down	tumor suppressor	Migration and invasion	[22]
	Colorectal cancer	down	tumor suppressor	Proliferation, migration and invasion	[23]
miR-708-5p	NSCLC	up	oncomiR	Proliferation, migration and invasion	[24]
	Colorectal cancer	up	oncomiR	Cell growth and invasion	[25]
	Bladder cancer	up	oncomiR	Apoptosis	[26]
	Breast cancer	down	tumor suppressor	Invasion, EMT	[27]
	Hepatocellular carcinoma	down	tumor suppressor	Proliferation, migration and invasion	[28,29]
	Gastric cancer	down	tumor suppressor	Proliferation and invasion	[30]
	NSCLC	down	tumor suppressor	Invasion, apoptosis	[31]
	Prostate cancer	down	tumor suppressor	Migration, invasion and apoptosis	[32]

TT—tumor tissue, NT—normal tissue.

## Data Availability

Datasets used in this study are available in the following repositories (https://ualcan.path.uab.edu/analysis.html, accessed on 20 October 2025), (https://oncodb.org/; accessed on 20 October 2025), (http://www.bioinfo-zs.com/smartapp/, accessed on 20 October 2025).

## Abbreviations

The following abbreviations are used in this manuscript:

ARMC5	Armadillo Repeat-Containing Protein 5
GSK3B	Glycogen Synthase-3 Beta
LINE2	Long Interspersed Nuclear Elements 2
*LPP*	Lipoma
LUAD	Lung Adenocarcinoma
LUSC	Lung Squamous Cell Carcinoma
miRNA	microRNA
mRNA	messenger RNA
MTSS1	Metastasis Suppressor Protein 1
NSCLC	Non-Small Cell Lung Carcinoma
NUAK1	SNF1-Like Kinase 1
SIRT3	Sirtuin 3
TE	Transposable Elements
*TENM4*	Teneurin Transmembrane Protein 4
TSGs	Tumor Suppressor Genes
YPEL3	Yippee Like 3
